# Development and Validation of a Nomogram for Assessing Survival in Patients With Metastatic Lung Cancer Referred for Radiotherapy for Bone Metastases

**DOI:** 10.1001/jamanetworkopen.2018.3242

**Published:** 2018-10-12

**Authors:** Wing-Keen Yap, Ming-Chieh Shih, Chin Kuo, Ping-Ching Pai, Wen-Chi Chou, Kai-Ping Chang, Mu-Hung Tsai, Ngan-Ming Tsang

**Affiliations:** 1 Department of Radiation Oncology, Linkou Chang Gung Memorial Hospital Medical Center, Taoyuan City, Taiwan; 2 Institute of Epidemiology and Preventive Medicine, College of Public Health, National Taiwan University, Taipei, Taiwan; 3 Department of Radiation Oncology, National Cheng Kung University Hospital, College of Medicine, National Cheng Kung University, Tainan, Taiwan; 4 Division of Medical Oncology, Department of Internal Medicine, Linkou Chang Gung Memorial Hospital, Taoyuan City, Taiwan; 5 Department of Otorhinolaryngology, Head, and Neck Surgery, Linkou Chang Gung Memorial Hospital, Taoyuan City, Taiwan; 6 Chang Gung University, Taoyuan City, Taiwan; 7 School of Traditional Chinese Medicine, Chang Gung University, Taoyuan City, Taiwan

## Abstract

**Importance:**

A survival prediction model for patients with bone metastases arising from lung cancer would be highly valuable.

**Objective:**

To develop and validate a nomogram for assessing the survival probability of patients with metastatic lung cancer receiving radiotherapy for osseous metastases.

**Design, Setting, Participants:**

In this prognostic study, the putative prognostic indicators for constructing the nomogram were identified using multivariable Cox regression analysis with backward elimination and model selection based on the Akaike information criterion. The nomogram was subjected to internal (bootstrap) and external validation; its calibration and discriminative ability were evaluated with calibration plots and the Uno C statistic, respectively. The training and validation set cohorts were from a tertiary medical center in northern Taiwan and a tertiary institution in southern Taiwan, respectively. The training set comprised 477 patients with metastatic lung cancer who received radiotherapy for osseous metastases between January 2000 and December 2013. The validation set comprised 235 similar patients treated between January 2011 and December 2017. Data analysis was conducted May 2018 to July 2018.

**Main Outcomes and Measures:**

The nomogram end points were death within 3, 6, and 12 months.

**Results:**

Of 477 patients in the training set, 292 patients (61.2%) were male, and the mean (SD) age was 62.86 (11.66) years. Of 235 patients in the validating set, 113 patients (48.1%) were male, and the mean (SD) age was 62.65 (11.49) years. In the training set, 186 (39%), 291 (61%), and 359 (75%) patients died within 3, 6, and 12 months, respectively, and the median overall survival was 4.21 (95% CI, 3.68-4.90) months. In the validating set, 84 (36%), 120 (51%), and 144 (61%) patients died within 3, 6, and 12 months, respectively, and the median overall survival was 5.20 (95% CI, 4.07-7.17) months. Body mass index (18.5 to <25 vs ≥25: hazard ratio [HR], 1.42; 95% CI, 1.14-1.78 and <18.5 vs ≥25: HR, 2.31; 95% CI, 1.56-3.44), histology (non–small cell vs small cell lung cancer: HR, 0.59; 95% CI, 0.41-0.86), epidermal growth factor receptor mutation (positive vs unknown: HR, 0.66; 95% CI, 0.46-0.93 and negative vs unknown: HR, 0.98; 95% CI, 0.66-1.45), smoking status (ever smoker vs never smoker: HR, 1.50; 95% CI, 1.24-1.83), age, and neutrophil to lymphocyte ratio were incorporated. The HRs of age and neutrophil to lymphocyte ratio were modeled nonlinearly with restricted cubic splines (both *P* < .001). The nomogram’s discriminative ability was good in the training set (C statistic, ≥0.77; *P* < .001) and was validated using both an internal bootstrap method (C statistic, ≥0.76; *P* < .001) and an external validating set (C statistic, ≥0.75; *P* < .001). The calibration plots for the end points showed optimal agreement between the nomogram’s assessment and actual observations.

**Conclusions and Relevance:**

The nomogram (with web-based tool) can be useful for assessing the probability of survival at 3, 6, and 12 months in patients with metastatic lung cancer referred for radiotherapy to treat bone metastases, and it may guide radiation oncologists in treatment decision making and engaging patients in end-of-life discussions and/or hospice referrals at appropriate times.

## Key Points

**Question:**

What are the optimal factors to use for constructing a nomogram to assess the probability of survival in patients with bone metastases arising from lung cancer?

**Findings:**

This prognostic study of 477 patients with metastatic lung cancer found that age, histological type, smoking status, epidermal growth factor receptor mutation status, body mass index, and neutrophil to lymphocyte ratio were ideal parameters to construct a nomogram for potentially indicating 3-, 6-, and 12-month survival. This nomogram was successfully validated in a separate cohort of 235 patients at another institution.

**Meaning:**

The study’s nomogram can guide radiation oncologists in making treatment decisions and determining the proper timing for end-of-life discussions and/or hospice referrals.

## Teaser

This prognostic study develops and validates a nomogram to assess the probability of survival at 3, 6, and 12 months in patients from Taiwan with metastatic lung cancer who were referred for radiotherapy to treat osseous metastases.

## Introduction

Lung cancer is the most commonly diagnosed malignant neoplasm and the leading cause of cancer-related death, with an incidence surpassing 1.6 million cases (approximately 13% of cancer diagnosed) and a mortality rate of approximately 1.4 million (reflecting 18% of cancer-related deaths) annually.^1^ A recent analysis of the Oncology Services Comprehensive Electronic Records database, which encompasses 569 000 patients from 52 cancer centers in the United States, revealed that lung cancer is only second to prostate cancer in the 10-year cumulative incidences of bone metastasis at a rate of 12.9% (95% CI, 12.6%-13.2%).^2^ Most patients who develop bone metastases will eventually receive radiotherapy for various reasons such as palliation for severe bone pain, spinal cord compression, prevention of pathological fracture or neurological deficit, or even long-term tumor control.^3,4^ Thus, patients receiving radiotherapy for bone metastases arising from lung cancer compose a large proportion of palliative service recipients in radiation oncology departments.

It is challenging for radiation oncologists who administer palliative radiotherapy to assess the life expectancies of patients with metastatic cancers because they can vary from days to years. Studies have shown that physicians’ clinical assessments of the survival of patients with terminal cancer are inaccurate and tend to be overly optimistic.^5,6,7^ Nevertheless, an accurate assessment of average survival in this population is of great importance for making critical decisions in daily clinical practice, such as individualized tailoring of treatment intensity (eg, the necessity of radiotherapy, radiopharmaceuticals, and/or other novel drugs; radiotherapy fractionation schema; and benefit of adding surgical interventions), when to enroll in hospice (as many countries require an estimated survival time of less than 6 months for hospice referrals), and design of and enrollment in clinical trials (because most clinical trials require an estimated survival of more than 3 months for eligibility).

Various prognostic models have been developed to help oncologists assess the life expectancy of patients with terminal cancer, but all models have limitations in terms of their ease of use, accuracy, and applicability to specific patient populations. A recent review concluded that none of the existing models could accurately assess the life expectancy of a spectrum of patients with end-stage cancer.^8,9,10,11,12^ Most of these models were developed based on patients with general advanced cancer and have not been validated for specific patient populations, including those with bone metastasis from lung cancer. Thus, these models might not be as generally accurate as presented during publication. While the number of risk factors (NRF) model by Chow et al^13^ has been externally validated in patients with bone metastasis arising from breast or prostate cancer, studies have shown that patients with metastatic lung cancers have distinctly worse prognoses than those with metastatic breast and prostate cancers.^9,10,14^ Therefore, a model specific to patients with bone metastasis arising from lung cancer is currently lacking.

In recent years, nomograms have been widely accepted in the oncology community as reliable tools for assessing patients’ prognoses. In this study, we aimed to develop and validate a nomogram for assessing the survival probability at 3, 6, and 12 months in patients with metastatic lung cancer referred for radiotherapy to treat bone metastases.

## Methods

### Patient Selection

Data from all patients with metastatic lung cancer who were referred for radiotherapy to treat bone metastases at the Linkou Chang Gung Memorial Hospital, a tertiary medical center in northern Taiwan, between January 2000 and December 2013, were used to develop the nomogram. All primary cancers were histologically confirmed. Patients with no height, weight, or complete blood cell count (with differential) measured within 2 weeks before initiating radiotherapy were excluded, as were those aged younger than 18 years at initial radiotherapy consultation. A different cohort of consecutive patients with metastatic lung cancer who received radiotherapy for bone metastases at the National Cheng Kung University Hospital, a tertiary medical center in southern Taiwan, between January 2011 and December 2017, composed the independent external validating set; their inclusion and exclusion criteria were the same as those for the training set.

The institutional review boards of both Linkou Chang Gung Memorial Hospital and Linkou Chang Gung Memorial Hospital approved this study. All participants included in the study signed general informed consent forms to allow their medical records and biological samples to be used for research purposes.

### Study Design

This study is reported in accordance with the Transparent Reporting of a Multivariable Prediction Model for Individual Prognosis or Diagnosis (TRIPOD) reporting guideline statement checklist for prediction model development and validation. The nomogram end points were death within 3, 6, and 12 months from the date of commencement of first radiotherapy to treat any bone metastasis. The presumptive nomogram prognosticators were clinical and pathological parameters selected a priori that correlated with prognoses of patients with metastatic cancers, including age at initial radiotherapy consultation, body mass index (BMI; calculated as weight in kilograms divided by height in meters squared), neutrophil to lymphocyte ratio (NLR), sex, histology, epidermal growth factor receptor (EGFR) mutation status, presence of non–bone metastasis, performance status, and smoking history.^9,10,15,16,17,18,19^ All data were retrospectively collected from cancer registries and electronic medical records from the 2 institutions. A senior radiation oncologist at each institution supervised the data collection process.

Neutrophil to lymphocyte ratio was derived from the complete blood cell count (with differential) obtained as part of routine blood tests. All measurements were obtained within 2 weeks before initiating radiotherapy and were categorized using standard cutoffs to define normal (<3), moderate (3 to <5), and high (≥5) inflammation.^16^ The World Health Organization’s BMI classification system was used to define underweight (<18.5), normal weight (18.5 to <25), overweight (≥25 to <30), and obese (≥30). Cigarette smoking status before the diagnosis of lung cancer was collected using a questionnaire on the date of first consultation with a radiation oncologist and was categorized according to the US Centers of Disease Control and Prevention classification system as never smoker (someone who smoked <100 cigarettes/lifetime) or ever smoker (someone who smoked ≥100 cigarettes/lifetime).

### Statistical Analysis

Multivariable Cox regression analysis was performed to calculate the hazard ratios and 95% confidence intervals of the putative prognosticators and build the nomogram. Categorical prognostic indicators (sex, histology, EGFR mutation status, presence of non–bone metastasis, performance status, and smoking history) were entered as dummy variables. Continuous prognostic indicators (age, BMI, and NLR) were categorized using existing aforementioned cutoffs, entered linearly or modeled using restricted cubic splines with 3, 4, or 5 knots. Restricted cubic splines assume that the effect of the prognostic indicator on the outcome is a smooth piecewise cubic polynomial with linear tails. They provide flexibility in fitting highly curved relationships, avoid heavy influence from outlying prognostic indicators, and may provide better power than dichotomizing continuous variables.^20,21^ A greater number of knots implies additional flexibility of the fitted curve. The positions of the knots were set as the 10th, 50th, and 90th percentiles with 3 knots; the 5th, 35th, 65th, and 95th percentiles with 4 knots; and the 5th, 27.5th, 50th, 72.5th, and 95th percentiles with 5 knots.^22^ For each setting combination for continuous variables, we generated a candidate model using backward elimination with Akaike information criterion (AIC) as the selection criterion.^23^ We selected the model with the lowest AIC as the final prediction model. The performance of the model was evaluated with calibration plots (which determine the agreement between the observed and estimated survival probability) and validation indices (which determine the discriminative ability of the models). Biased-corrected internal calibration was performed at 3, 6, and 12 months using 400 bootstrap resamples and fitting the relationship between the observed and estimated survival probabilities with flexible spline hazard regression.^24^ The 95% quantile of the absolute prediction error (the absolute difference between the observed and estimated survival probability) was also calculated for numerical reference. For internal validation, Uno C statistics at 3, 6 and 12 months were calculated to represent discriminative ability.^25^ A C statistic significantly greater than 0.5 indicates good discrimination of the model.^25^ The merit of adopting Uno C statistic is its consistency in the presence of censored data, whereas the traditional Harrell C statistic may be sensitive to the mechanism of censoring.^25^ Bias-corrected internal validation was also performed by evaluating Uno C statistic under 1000 bootstrap resamples.^24^ External calibration and validation were performed using the same methods on the validating data set. Based on the fitted prediction model, we produced a corresponding nomogram^24^ and an interactive web-based survival probability application with Shiny, version 0.13.2.^26^ All analyses were performed with the Regression Modeling Strategies package, version 5.1-2 in R, version 3.3.2 (R Foundation).^24,27^

## Results

### Training Set

The training set included 477 eligible patients whose clinical and pathological characteristics are shown in Table 1. Of the 477 patients in the training set, 292 patients (61.2%) were male, and the mean (SD) age was 62.86 (11.66) years. After a median follow-up of 4.18 months (interquartile interval, 1.78-11.54 months), there were 186 (39%), 291 (61%), and 359 (75%) deaths within 3, 6, and 12 months, respectively. The median overall survival was 4.21 months (95% CI, 3.68-4.90 months). Body mass index (18.5 to <25 vs ≥25: hazard ratio [HR], 1.42; 95% CI, 1.14-1.78 and <18.5 vs ≥25: HR, 2.31; 95% CI, 1.56-3.44), histology (non–small cell vs small cell lung cancer: HR, 0.59; 95% CI, 0.41-0.86), epidermal growth factor receptor mutation (positive vs unknown: HR, 0.66; 95% CI, 0.46-0.93 and negative vs unknown: HR, 0.98; 95% CI, 0.66-1.45), smoking status (ever smoker vs never smoker: HR, 1.50; 95% CI, 1.24-1.83), age, and neutrophil to lymphocyte ratio were incorporated. The Kaplan-Meier curve is shown in eFigure 1A in the Supplement.

**Table 1.** Clinicopathological Characteristics of the Patients

Baseline Characteristics	No. (%)		P Value
	Training Set (n = 477)	Validating Set (n = 235)	
Time, y	2000-2013	2011-2017	
Age, mean (SD), y	62.86 (11.66)	62.65 (11.49)	.28
BMI, mean (SD)	23.05 (3.50)	22.26 (3.74)	.01
NLR, median (IQR)	4.46 (4.82)	6.59 (7.52)	<.001
Sex			
Female	185 (38.8)	122 (51.9)	.001
Male	292 (61.2)	113 (48.1)	
Categorized age, y			
<60	194 (40.7)	98 (41.7)	.70
60-70	143 (30.0)	72 (3.6)	
>70	140 (29.4)	65 (27.7)	
Categorized BMI			
<18.5	37 (7.8)	55 (23.5)	<.001
18.5 to <25	327 (68.6)	142 (6.4)	
≥25	113 (23.7)	38 (16.2)	
Categorized NLR			
<3	133 (27.9)	37 (15.7)	<.001
3 to <5	130 (27.3)	49 (2.9)	
≥5	214 (44.9)	149 (63.4)	
Histology			
Small cell lung cancer	34 (7.1)	4 (1.2)	<.001
Squamous cell carcinoma	53 (11.1)	11 (6.7)	
Adenocarcinoma	266 (55.8)	133 (81.6)	
Others^a^	124 (26.0)	17 (1.4)	
EGFR mutation status			
Not tested	409 (85.7)	37 (15.7)	<.001
Tested	68 (14.3)	198 (84.3)	
Negative	28/68 (41.2)	69/198 (34.8)	
Positive	40/68 (58.8)	129/198 (65.2)	
Nonbone metastasis			
Absent	408 (85.5)	106 (45.1)	<.001
Present	69 (14.5)	129 (54.9)	
Performance status^b^			
KPS 70-100, ECOG 0-1	309 (64.8)	97 (41.3)	<.001
KPS 10-60, ECOG 2-4	168 (35.2)	138 (58.7)	
Smoking history			
Never smoker	239 (50.1)	149 (63.4)	.001
Ever smoker	238 (49.9)	86 (36.6)	

Abbreviations: BMI, body mass index (calculated as weight in kilograms divided by height in meters squared); ECOG, Eastern Cooperative Oncology Group; EGFR, epidermal growth factor receptor; IQR, interquartile range; KPS, Karnofsky Performance Status; NLR, neutrophil to lymphocyte ratio.

^a^ Other histologies include large cell carcinoma, adenosquamous carcinoma, and non–small cell carcinoma not otherwise specified.

^b^ Possible KPS scores range from 0 to 100, with higher scores indicating better performance status (100 denoting perfect health and 0 denoting death). Possible ECOG scores range from 0 to 5, with higher scores indicating worse performance status (0 denoting perfect health and 5 denoting death).

**Figure 1.**
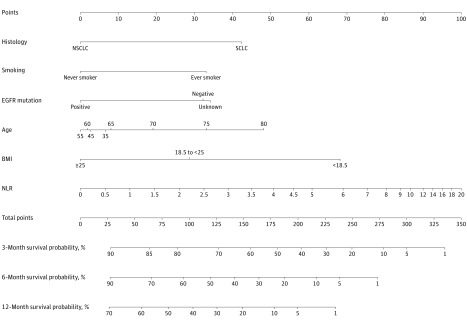
The Nomogram Developed in This Study The nomogram survival probability in patients with metastatic lung cancer referred for radiotherapy to treat bone metastases. A free web-based tool^28^ for using the nomogram is provided. BMI indicates body mass index (calculated as weight in kilograms divided by height in meters squared); EGFR, epidermal growth factor receptor; NLR, neutrophil to lymphocyte ratio; NSCLC, non–small cell lung cancer; and SCLC, small cell lung cancer.

Univariable analysis results are shown in Table 2. After backward elimination and model selection based on AIC, 6 prognostic indicators, including age, histology, smoking status, EGFR mutation status, BMI, and NLR, were included in the final model for the construction of the nomogram. Results of the final multivariable Cox regression model are shown in Table 3, where age and NLR were modeled nonlinearly with restricted cubic splines (both *P* < .001. The estimated spline function of age (eFigure 2A in the Supplement) indicated that the effect of this parameter on survival was “J” shaped, with the age of the lowest hazard being 55 years. The estimated spline function of NLR (eFigure 2B in the Supplement) showed that the hazardous effect of each NLR unit increase was relatively linear but was diminished above an NLR value of 9. Subtypes of non–small cell lung cancer were combined as 1 category in the final model because this model produced a lower AIC score compared with the model that incorporated different histological subtypes of non–small cell lung cancer (eTable 1 in the Supplement), and the differences in the hazard ratios among them were too small to significantly affect the nomogram. In addition, an interaction analysis was performed to evaluate the influence of the interactions between the possible effect modifiers (sex, histology, and age) and the variables in the final model on the performance of the final model. None of the interactions were statistically significant (eTable 2 in the Supplement), and all of them resulted in a lower AIC score when entered into the final model. The nomogram, along with a free web-based tool^28^ for survival probabilities in this particular population, is shown in Figure 1. By summing the points from each variable, locating the total points on the scale, and drawing a straight line down to the end point scales, the nomogram allows users to easily estimate the probability of survival at 3, 6, and 12 months. This web-based tool can be used for more precise calculations and also reports 95% prediction intervals; the assumed end point is adjustable up to 36 months.

**Table 2.** Univariable Analysis of Putative Clinicopathological Variables

Baseline Characteristics	Hazard Ratio (95% CI)	*P* Value
Age, y	1.01 (1.01-1.02)	<.001
BMI	0.95 (0.82-0.97)	<.001
NLR	1.02 (1.02-1.03)	<.001
Sex		
Female	1 [Reference]	
Male	1.69 (1.40-2.04)	<.001
Categorized age, y		
<60	1 [Reference]	
60-70	1.03 (0.82-1.28)	.79
>70	1.59 (1.28-1.99)	<.001
Categorized BMI		
<18.5	2.84 (1.94-4.16)	<.001
18.5 to <25	1.43 (1.15-1.78)	.001
≥25	1 [Reference]	
Categorized NLR		
<3	1 [Reference]	
3 to <5	1.23 (0.96-1.58)	.10
≥5	2.31 (1.84-2.89)	<.001
Histology		
Small cell lung cancer	1 [Reference]	
Non–small cell lung cancer	0.40 (0.28-0.57)	<.001
Squamous cell carcinoma	0.51 (0.33-0.79)	.003
Adenocarcinoma	0.36 (0.25-0.52)	<.001
Others^a^	0.46 (0.31-0.67)	<.001
EGFR mutation status		
Unknown	1 [Reference]	
Positive	0.60 (0.43-0.84)	.003
Negative	0.97 (0.66-1.42)	.86
Nonbone metastasis		
Absent	1 [Reference]	
Present	0.89 (0.69-1.15)	.38
Performance status^b^		
KPS 70-100, ECOG 0-1	1 [Reference]	
KPS 10-60, ECOG 2-4	1.26 (1.04-1.52)	.02
Smoking history		
Ever smoker	1 [Reference]	
Never smoker	1.79 (1.48-2.15)	<.001

Abbreviations: BMI, body mass index (calculated as weight in kilograms divided by height in meters squared); ECOG, Eastern Cooperative Oncology Group; EGFR, epidermal growth factor receptor; KPS, Karnofsky Performance Status; NLR, neutrophil to lymphocyte ratio.

^a^ Other histologies include large cell carcinoma, adenosquamous carcinoma, and non–small cell carcinoma not otherwise specified.

^b^ Possible KPS scores range from 0 to 100, with higher scores indicating better performance status (100 denoting perfect health and 0 denoting death). Possible ECOG scores range from 0 to 5, with higher scores indicating worse performance status (0 denoting perfect health and 5 denoting death).

**Table 3.** Final Model of the Multivariable Cox Regression Analysis

Terms	Hazard Ratio (95% CI)	*P* Value
Nonlinear terms		
Age, y		<.001
NLR		<.001
Categorical terms		
BMI		
≥25	1 [Reference]	
18.5 to <25	1.42 (1.14-1.78)	.002
<18.5	2.31 (1.56-3.44)	<.001
Histology		
Small cell lung cancer	1 [Reference]	
Non–small cell lung cancer^a^	0.59 (0.41-0.86)	.01
EGFR mutation status		
Unknown	1 [Reference]	
Positive	0.66 (0.46-0.93)	.02
Negative	0.98 (0.66-1.45)	.91
Smoking history		
Never smoker	1 [Reference]	
Ever smoker	1.50 (1.24-1.83)	<.001

Abbreviations: BMI, body mass index (calculated as weight in kilograms divided by height in meters squared); EGFR, epidermal growth factor receptor; NLR, neutrophil to lymphocyte ratio.

^a^ Includes squamous cell carcinoma, adenocarcinoma, large cell carcinoma, adenosquamous carcinoma, and non–small cell carcinoma not otherwise specified.

**Figure 2.**
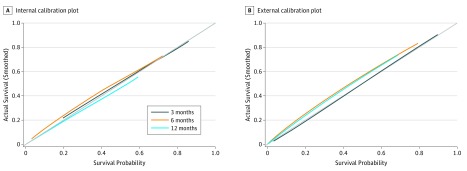
Calibration Plots for Estimating Survival Probability at 3, 6, and 12 Months Calibration plots are shown for the training cohort (A) and the external validating cohort (B). The 45° gray line is the reference line that indicates where a perfect calibration would lie.

The calibration plot for internal validation showed excellent agreement of 3-, 6-, and 12-month survival probabilities between the nomogram’s estimated and actual observations (Figure 2A). The 95% quantiles of the absolute prediction error were 2.02% for 3 months, 4.29% for 6 months, and 3.77% for 12 months; ie, 95% of the absolute prediction errors on the observed probabilities of survival of the participants having the same total scores on the nomogram were less than 5% for the points at 3, 6, and 12 months. In terms of discriminative ability, Uno C statistic on the training set was 0.77 (95% CI, 0.73-0.81; *P* < .001) for 3 months, 0.78 (95% CI, 0.73-0.82; *P* < .001) for 6 months, and 0.80 (95% CI, 0.75-0.85; *P* < .001) for 12 months. Bootstrap validation showed that the optimism-corrected C statistic was 0.76 (95% CI, 0.72-0.80; *P* < .001) for 3 months, 0.77 (95% CI, 0.72-0.81; *P* < .001) for 6 months, and 0.79 (95% CI, 0.74-0.84; *P* < .001) for 12 months, which indicated good discrimination.

### Validating Set

The validating set included 235 eligible patients whose clinicopathological characteristics are summarized in Table 1. Of the 235 patients in the validating set, 113 patients (48.1%) were male, and the mean (SD) age was 62.65 (11.49) years. After a median follow-up of 4.10 months (interquartile interval, 1.52-11.45 months), there were 84 (36%), 120 (51%), and 144 (61%) deaths within 3, 6, and 12 months, respectively. The median overall survival was 5.20 months (95% CI, 4.07-7.17 months).

The calibration plot for external validation (Figure 2B) showed good agreement between the nomogram’s estimated and the observed probabilities of survival at 3, 6, and 12 months. The 95% quantiles of the absolute prediction error was 0.85% for 3 months, 6.44% for 6 months, and 5.37% for 12 months; ie, 95% of the absolute prediction errors on the observed probabilities of survival of the participants (in the validating set) with the same total scores on the nomogram were less than 7% for the points at 3, 6, and 12 months. Uno C statistic for the established nomogram in the validating cohort was 0.75 (95% CI, 0.68-0.82; *P* < .001) for 3 months, 0.77 (95% CI, 0.71-0.84; *P* < .001) for 6 months, and 0.77 (95% CI, 0.70-0.84; *P* < .001) for 12 months, which showed that this nomogram remained valid for use in an external cohort.

To compare the performance of our nomogram against the existing Chow’s NRF model,^9,13^ which is a palliative prognostic tool developed using more patients with general metastatic cancer in a palliative radiotherapy setting, an NRF score was generated for each patient in the validating set according to the number of risk factors (non-breast primary cancer [ie, lung cancer], presence of metastases other than bone metastases, and Karnofsky Performance Status of ≤60). Uno C statistic for this model was 0.58 (95% CI, 0.51-0.66; *P* = .03) for 3 months, 0.58 (95% CI, 0.51-0.66; *P* = .03) for 6 months, and 0.59 (95% CI, 0.50-0.67; *P* = .05) for 12 months. Thus, our model outperforms Chow’s NRF model by absolute increments of the C statistic of 0.17 (95% CI, 0.06-0.27; *P* < .001) for 3 months, 0.19 (95% CI, 0.10-0.29; *P* < .001) for 6 months, and 0.18 (95% CI, 0.08-0.28; *P* < .001) for 12 months.

## Discussion

Patients who develop bone metastases from lung cancer have grave prognoses; survival is typically measured in the months following the detection of metastases.^14^ However, great variability still exists in terms of life expectancy in this population, making survival particularly difficult to assess and causing some patients to be subjected to futile, prolonged, and/or aggressive therapies that may be more physically and financially detrimental than beneficial while unnecessarily depleting resources.^29^ A Surveillance, Epidemiology, and End Results study in the United States found that approximately 20% of patients with terminal cancer spent 10 of their last 30 days of life receiving radiotherapy.^30^ Another study of 216 patients showed similar findings, half of those patients spent more than 60% of their remaining days receiving palliative radiotherapy.^31^ In theory, these unwanted circumstances could be reduced by accurately estimating survival that can help tailor treatments, eg, by using short-course radiotherapy, avoiding surgical intervention, and/or avoiding expensive radiopharmaceutical therapies and other novel drugs for patients with bone metastasis with less than 3 months of life expectancy. Additionally, our nomogram can be used for designing clinical trials for patients with bone metastases arising from lung cancer and for identifying such patients who are eligible for enrollment in trials of novel therapies.

Radiation oncologists play an important role in the palliative care of patients with cancer and should be actively involved in end-of-life discussions, including initiating hospice referrals when appropriate. However, physicians tend to lack confidence in estimating life expectancy,^32^ making them more likely to withhold prognostic information from their patients and to miss the optimal timing of conducting end-of-life discussions and/or providing hospice referrals.^33^ Furthermore, from the patients’ perspective, studies showed that approximately 80% of patients with metastatic cancers wished to be informed of the average survival rates of patients with their type of disease,^34^ and those with end-stage cancers who were involved in end-of-life discussions were more likely to avoid aggressive medical care that was associated with a lower quality of life, higher medical care costs, and worse caregiver bereavement outcomes.^35,36,37^ Thus, with 3 clinically valuable end points (ie, 3, 6, and 12 months), our nomogram not only could assist radiation oncologists in making critical treatment decisions but could also provide much appreciated and important survival information to patients, thereby allowing shared decision making that is more patient oriented.

While age, NLR, BMI, histology, EGFR mutation status, and smoking history were selected for the estimation of survival probability at 3 time points, performance status was eliminated from the final model through a model selection process even though this variable has been previously identified as a significant prognostic factor.^9,10,32^ Although performance status was a significant prognosticator on univariable analysis, this factor was eliminated because its effect on estimating survival was outweighed by the effect of BMI and NLR on multivariable analyses. Both BMI and NLR are associated with performance status because BMI acts as a surrogate for poor nutritional status and cancer-attributable cachexia, while NLR reflects systemic inflammation that is also an important driver of cancer-related cachexia.^38,39^ Recent studies have consistently found that BMI is significantly associated with survival both in patients with nonmetastatic cancers^18,40,41^ and in those with metastases.^15,42^ Similarly, NLR has been shown to be a strong indicator of survival in patients with cancer.^16,41^ Of note, a recent study of patients with lung or gastrointestinal cancer by Martin et al^43^ reported that models containing only cachexia-related factors (BMI, weight loss, muscle index, and muscle attenuation) outperformed those encompassing conventional variables (cancer type, stage, age, and performance status). Their findings concurred with ours in that replacing the more subjective performance status score with objective cancer cachexia-related factors, such as BMI and NLR, produced a better-performing model. Although some studies have shown that sarcopenia is a better marker for cancer-related cachexia than BMI, we chose the latter for the nomogram because of its ease of measurement in routine clinical settings. Similarly, in this study, sex was a significant prognosticator on univariable analysis but was eliminated from the final model; further analysis found that its predictive effect diminished with the inclusion of EGFR mutation status in the model. This might be explained by the fact that Asian female patients with lung cancer are more likely to have adenocarcinoma with EGFR mutation,^44,45^ making the disease biologically distinct and more effectively treatable with targeted therapy. Thus, the favorable prognostic effect of female sex actually stems from women’s greater likelihood of being EGFR mutation positive.

### Limitations

The discriminative ability of our nomogram in the training set was reproduced in the independent validating set. However, for calibration, our nomogram slightly underestimated the observed probability of survival at 6 and 12 months when applied to the validating set. As the points on the calibration plot are parallel to the reference line, we posit that this small underestimation in the external calibration result was mainly owing to the longer survival of patients in the validating set than in the training set and was not due to a difference in the prognostic variables. The longer survival of patients in the validating set was probably because these patients had more favorable profiles in terms of baseline clinicopathological characteristics (Table 1) and were treated more recently, where advancements in patients care and treatment strategies may have improved their survival. Nevertheless, the discrepancy is minor and would not impede the nomogram’s use in contemporary clinical practice. Notably, we selected a cohort of recently treated patients for inclusion in the external validating set with the specific goal of providing a temporal validation for our nomogram. Thus, such differences in the baseline characteristics between the training set and the validating set are intended to reflect the temporal, demographic, and geographic variations in cancer epidemiology and treatment approach in the real world.

Method limitations of our study include its retrospective nature and the ethnically homogeneous patient population. A prospective study in Asian patients with advanced lung adenocarcinoma found that Taiwanese patients have an EGFR mutation rate of around 60% (comparable with the rates reported in this study), which is much higher than the rates reported in Japanese patients (approximately 30%) and white populations (approximately 20%), and the rates vary significantly even among Asian countries.^46^ Of note, family history of lung cancer and environmental risk factors such as secondhand smoke, household air pollution, and tuberculosis exposure were found to be associated with an increased risk of lung cancer in Taiwanese nonsmokers in a recent prospective nationwide study.^47^ The environmental and genetic influences on the etiology of carcinogenesis and the biology of cancer in different ethnic populations might be different and hence, may limit the applicability of our model across different ethnic groups. In addition, excluding patients with height, weight, or complete blood cell count (with differential) not measured within 2 weeks before initiating radiotherapy from this study might have introduced a selection bias and limited its generalizability. Therefore, our data should receive longitudinal validation among ethnically diverse patients.

## Conclusions

We developed and validated a nomogram to estimate the probability of survival at 3, 6, and 12 months in patients with metastatic lung cancer who were referred for radiotherapy to treat osseous metastases. Based on 6 objective and easy-to-acquire variables, our nomogram and free web-based tool provided clinically relevant information to patient and can assist radiation oncologists in tailoring treatment approaches to the patients’ life expectancies, including initiating end of life discussions and/or hospice referrals at appropriate times.

## Notes

Supplement. **eFigure 1.** Kaplan-Meier Curves for Overall Survival (A) in the Training Set and (B) in the Validating Set **eFigure 2. **Hazard Ratio Charts for Age and Neutrophil-to-Lymphocyte Ratio (NLR) **eTable 1.** Multivariable Cox Regression Model (with different subtypes of NSCLC) **eTable 2.** Interaction Analysis of the Possible Effect Modifiers
