# Supplementary material for: Development and Validation of a Nomogram for Assessing Survival in Patients With Metastatic Lung Cancer Referred for Radiotherapy for Bone Metastases
Source: JAMA Netw Open. 2018 Oct 12;1(6):e183242. doi: 10.1001/jamanetworkopen.2018.3242 (PMC6324455; doi:10.1001/jamanetworkopen.2018.3242)
Supplement: Supplement. — eFigure 1. Kaplan-Meier Curves for Overall Survival (A) in the Training Set and (B) in the Validating Set eFigure 2. Hazard Ratio Charts for Age and Neutrophil-to-Lymphocyte Ratio (NLR) eTable 1. Multivariable Cox Regression Model (with different subtypes of NSCLC) eTable 2. Interaction Analysis of the Possible Effect Modifiers [file jamanetwopen-1-e183242-s001.pdf]

Supplementary Online Content

Yap W-K, Shih M-C, Kuo C, et al. Development and validation of a nomogram for assessing survival in patients with metastatic lung cancer referred for radiotherapy for bone metastases. *JAMA Netw Open*. 2018;1(6):e183242. doi:10.1001/jamanetworkopen.2018.3242

eFigure 1. Kaplan-Meier Curves for Overall Survival (A) in the Training Set and (B) in the Validating Set

eFigure 2. Hazard Ratio Charts for Age and Neutrophil-to-Lymphocyte Ratio (NLR)

eTable 1. Multivariable Cox Regression Model (with different subtypes of NSCLC)

eTable 2. Interaction Analysis of the Possible Effect Modifiers

This supplementary material has been provided by the authors to give readers additional information about their work.

© 2018 Yap W-K et al. *JAMA Network Open*.

eFigure 1. Kaplan-Meier Curves for Overall Survival (A) in the Training Set and (B) in the Validating Set

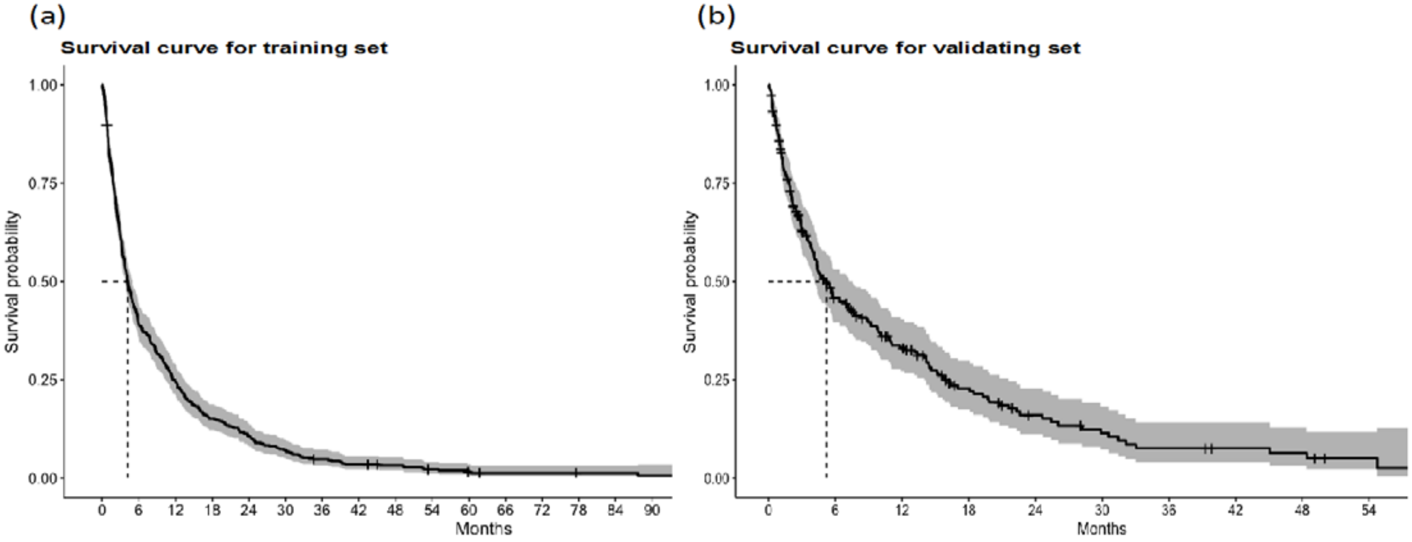

© 2018 Yap W-K et al. *JAMA Network Open*.

eFigure 2. Hazard Ratio Charts for Age and Neutrophil-to-Lymphocyte Ratio (NLR)

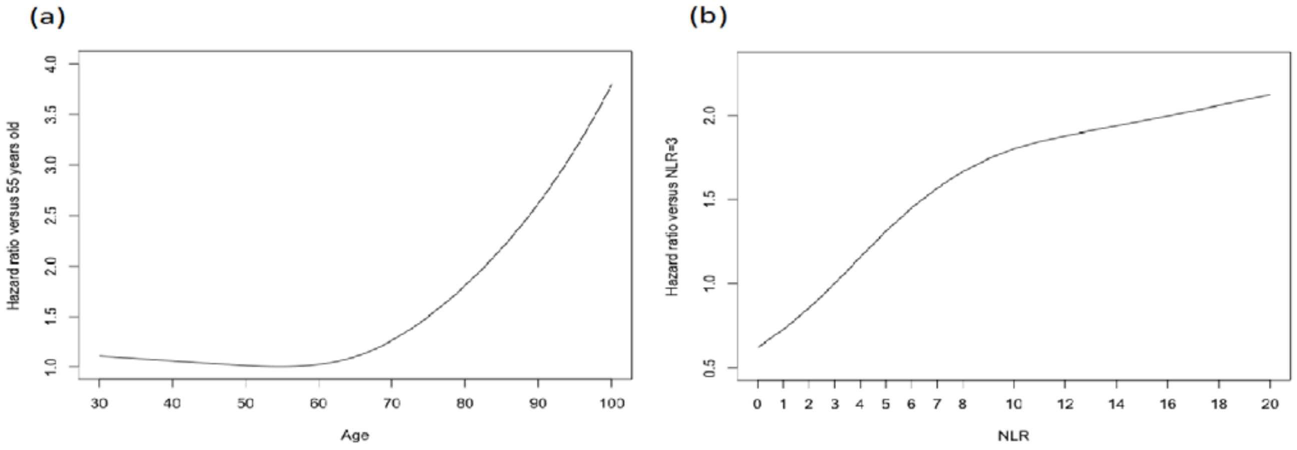

Non-linear relationship between continuous prognosticators and hazard showing (a) age at initial radiotherapy consultation and (b) NLR.

© 2018 Yap W-K et al. *JAMA Network Open*.

eTable 1. Multivariable Cox Regression Model (with different subtypes of NSCLC)

	Hazard ratio (95% CI)		P-value
**Non-linear terms**			
**Age (years)**	-		<0.001
**NLR**	-		<0.001
**Categorical terms**			
**BMI (kg/m^2)**			
**≥25**	ref.		
**18.5 to <25**	1.42	(1.14–1.79)	0.002
**<18.5**	2.32	(1.56–3.47)	<0.001
**Histology**			
**Small cell lung cancer**	ref.		
**Squamous cell carcinoma**	0.58	(0.37–0.91)	0.018
**Adenocarcinoma**	0.59	(0.39–0.88)	0.009
**Others[a]**	0.60	(0.41–0.90)	0.012
***EGFR* mutation status**			
**Negative**	ref.		
**Positive**	0.67	(0.41–1.11)	0.12
**Unknown**	1.02	(0.68–1.52)	0.94
**Smoking history**			
**Never smoker**	ref.		
**Ever smoker**	1.50	(1.24–1.83)	<0.001

Abbreviations: BMI, body mass index; NLR, neutrophil-to-lymphocyte ratio; EGFR, epidermal growth factor receptor; 95% CI, 95% confidence interval; ref., reference category; NSCLC, non-small cell lung cancer.

[a]Other histologies include large cell carcinoma, adenosquamous carcinoma, and non-small cell carcinoma not otherwise specified.

© 2018 Yap W-K et al. *JAMA Network Open*.

eTable 2. Interaction Analysis of the Possible Effect Modifiers[a]

	Effect modifiers		
	Sex	Histology	Age
Histology	NA[b]	-	0.24
EGFR mutation	0.14	NA[c]	0.09
Smoking	0.31	0.28	0.89
Age	0.54	0.51	-
BMI	0.66	0.79	0.17
NLR	0.59	0.28	0.11

Abbreviations: BMI, body mass index; NLR, neutrophil-to-lymphocyte ratio; EGFR, epidermal growth factor receptor.

[a]The numbers in the table are p-values.

[b]Sex-histology interaction is not estimable due to lack of female SCLC patients.

[c]Histology-EGFR interaction is not estimable because SCLC patients were not tested for EGFR mutation.

© 2018 Yap W-K et al. *JAMA Network Open*.
